# Caliber of sensory axons in vivo varies spatially and temporally and is influenced by the cellular microenvironment

**DOI:** 10.1101/2024.12.04.626901

**Published:** 2024-12-09

**Authors:** Kaitlin Ching, Alvaro Sagasti

**Affiliations:** 1 Department of Cell, Molecular, and Developmental Biology, University of California, Los Angeles, Los Angeles, California 90095, USA

## Abstract

Cell shape is crucial to cell function, particularly in neurons. The cross-sectional diameter, also known as caliber, of axons and dendrites is an important parameter of neuron shape, best appreciated for its influence on the speed of action potential propagation. Most studies of axon caliber focus on cell-wide regulation and assume that caliber is static. Here, we have investigated local variation and dynamics of axon caliber in the peripheral axons of zebrafish touch-sensing neurons at embryonic stages, prior to sex determination. To obtain absolute measurements of caliber in vivo, we paired sparse membrane labeling with super-resolution microscopy of neurons in live fish. We found that axon segments had varicose or “pearled” morphologies, and thus vary in caliber along their length, consistent with reports from mammalian systems. Sister axon segments originating from the most proximal branch point in the axon arbor had average calibers that were largely independent of each other. Axon caliber tapered across the branch point, suggesting that action potential conductance may be favored in these afferent axons. Caliber was dynamic on the time-scale of minutes, and this dynamicity changed over the course of development. By measuring the caliber of axons adjacent to dividing epithelial cells, we found that the cellular microenvironment is one of potentially multiple drivers of axon caliber variation across space and time. Our findings raise the possibility that spatial and temporal variation in axon caliber could significantly influence neuronal physiology.

## Introduction

Across biological systems, branched architecture allows for coverage of large regions. includes plant root systems, animal circulatory and respiratory systems, and fungal hyphae (Ochoa-Espinosa and Affolter, 2012). However, much remains unexplored about regulation at specific locations in such a broad network, particularly when that branched network is within a single cell, such as a neuron.

**Figure 1:**
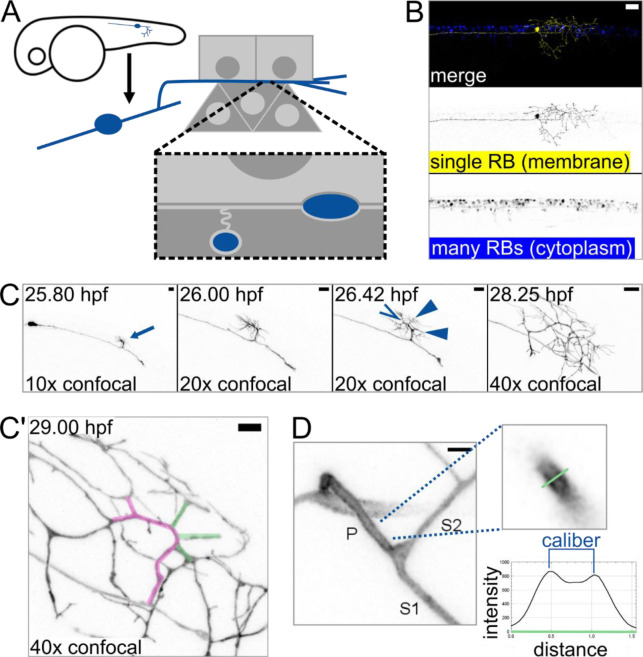
Axon branch development and caliber in sparsely-labeled Rohon-Beard (RB) neurons **(A)** Diagram depicting an RB neuron in an embryonic zebrafish. Light grey squares represent periderm (outermost) epithelial cells. Dark grey triangles represent basal epithelial cells, which ensheath some RB axons around 2 – 3 dpf. **(B)** Example of embryonic zebrafish tail with sparse labeling showing one RB neuron expressing EGFP-CAAX among many RB neurons expressing cytoplasmic DsRed. Scale bar: 50 μm. **(C)** Illustrative images taken from time lapse of RB neuron development. Arrow: formation of main branch in peripheral axon arbor. 26.42 hpf panel shows multiple new branches that formed within minutes of each other. Open arrowhead: growth cone bifurcation event. Solid arrowheads: collateral sprouting events. Scale bars: 10 μm. **(C’)** Proximal region of the axon arbor shown in B. Magenta pseudo-colored axon shows the main branch, which branched by growth cone bifurcation. Green pseudo-colored axons show branches that formed by subsequent collateral sprouting. Scale bar: 5 μm. **(D)** Method used for measuring caliber. RB neurons expressing EGFP-CAAX were measured by line scan (green line). Caliber was scored as distance between intensity peaks, as illustrated in example plot. P: primary branch, which extends from spinal cord to skin, S1: thicker secondary branch, S2: thinner secondary branch. Scale bar: 2 μm.

The shape of a neuron’s axons or dendrites, like the shape of all cells, is central to its function. A key characteristic of that shape is cross-sectional diameter, known as caliber. Axon caliber intrinsically influences the speed of action potential propagation (Sakaguchi et al., 1993; Kriz et al., 2000) and may also play a role in cell health and structural stability (Perge et al., 2012; Dollé et al., 2018). One example of an axon with branched architecture is the skin-innervating peripheral arbor of a Rohon-Beard (RB) neuron, a type of touch-sensing neuron that develops in early embryonic zebrafish (Fig 1A) (Wang et al., 2013; Katz et al., 2021). RB peripheral arbors are afferent axons that detect somatosensory stimuli in the skin (Shorey et al., 2021). RB neurons then relay messages to interneurons via central axons in the spinal cord, thus promoting behavioral responses.

The significance of axon caliber in cell function has been described for about a century (Hursh, 1939). It is widely regarded as a cell-wide trait despite reports of variations in caliber along the length of axons and dendrites. Here, we use zebrafish RB neurons as a model to characterize axon caliber. We found that a single cell in vivo can have a variety of axon calibers, varying within a segment and between sister segments. Axon calibers were dynamic on the time-scale of minutes, and these dynamics changed over the developmental time-scale of days. Finally, we found that the extracellular environment impacts axon caliber, highlighting the complexities of the in vivo axon environment.

## Materials and Methods

### Fish care

Zebrafish (Danio rerio) adults were housed at 28.5°C with 13.5/10.5-hour light/dark cycles. Embryos were raised at 28.5°C in water containing 0.06 g/L Instant Ocean salt mix (Spectrum Brands, AA1–160P) and 0.05% methylene blue (ThermoFisher, 042771.AP).

### Injection for sparse labeling

To sparsely label RB neurons for observation of development and axon caliber, adult fish expressing Isl1[ss]:Gal4, UAS:dsRed (Sagasti et al., 2005) were crossed, and resulting embryos were allowed to develop for approximately one hour after fertilization. When embryos reached the 4-cell or 8-cell stage, a single cell in each embryo was injected with approximately 1–3 nL of plasmid containing the Gal4 effector, UAS:egfp-caax, diluted to 10–15 ng/μL in water, with or without phenol red (Sigma, P4758). After injection, embryos were raised as described above.

### Mounting embryos for microscopy

Unless otherwise indicated, embryos were immobilized on coverslips in 1.2% low-melt agarose dissolved in water (Fisher, BP165–25) and mounted with an equal volume of water containing 0.12 g/L Instant Ocean (Spectrum Brands, AA1–160P) and 0.4 mg/mL tricaine (MS-222, Western Chemical Inc.).

### Developmental time lapse imaging

Embryos injected for sparse labeling were sorted at approximately 20 hours post-fertilization (hpf) to find animals with a single cell expressing EGFP-CAAX in the tail. Selected embryos were immediately dechorionated using forceps and anesthetized in approximately 0.2 mg/mL tricaine (MS-222, Western Chemical Inc.). Embryos were mounted as described above. A cut was made in the agarose posterior to the tail using a fly pin probe to allow room for growth. Embryos were imaged at 5-minute intervals on a ZEISS laser scanning microscope (LSM) 800 with a stage heater at 28°C. Magnification was increased as needed by changing objectives to see branching. The most proximal branch point at approximately 28 hpf was identified and visually tracked back to the point of formation within the time lapse for scoring.

### Line scan and symmetry analysis

Embryos were injected for sparse labeling, as described above. Embryos with a distinct EGFP-CAAX-positive neuron in the tail were dechorionated using forceps and anesthetized in approximately 0.2 mg/mL tricaine (MS-222, Western Chemical Inc.). For this experiment, embryos were immobilized in 1.1 – 1.2% low-melt agarose on No.1 coverslips (Epredia, 12460S) to improve available working distance, and water used in initial anesthetization was added before sealing with a glass slide. The primary axon segment was identified as the one that exited the spinal cord and entered the skin. The most proximal branch point was imaged on a ZEISS LSM 880 with Airyscan deconvolution at 27 – 31 hpf. If two neurons or two arbors were present in the same animal or cell, the more posterior one was scored. Line scan analysis was performed using the Line and Plot Profile tools in ImageJ (Schindelin et al., 2012) to find intensity values along a line perpendicular to the center axis of the axon. Axon caliber was measured as the distance between the two peaks on the plot that corresponded to the plasma membrane. For symmetry analysis, axons were measured 3, 4, and 5 μm away from the branch point and averaged to find the caliber of the given segment. The caliber of secondary branches was normalized by dividing by the caliber of the corresponding primary branch. We defined symmetry as S2/S1, which is equivalent to the normalized S2 caliber divided by the normalized S1 caliber.

### Caliber dynamics time lapse imaging

Embryos were injected for sparse labeling, as described above. Around 24 hpf, embryos were mounted as described above. The proximal region of the axon arbor was imaged on a ZEISS LSM 980, heated to approximately 28°C, and processed with Airyscan Joint Deconvolution at a maximum of five iterations. Time lapses with 5-minute intervals were started at 28 – 31 hpf and acquired until the axon drifted out of the field of view, typically one to two hours. After imaging, embryos were recovered from the mounting agarose using forceps and a probe and grown an additional 24 hours under normal conditions (described above), with the addition of 0.2 mM PTU (1-phenyl 2-thiourea, Sigma, P7629) in the water. At 52 – 55.5 hpf, embryos were mounted and imaged in the same manner as the first day. For scoring, axon segment calibers were measured 3 μm from a branch point at every frame of the time lapse, and segments used were each separated by at least one branch point. If the same segments could be identified on the second day, caliber was scored at the same locations for every frame of the time lapse.

### Dual color time lapse imaging of cell division

To observe axons near dividing cells, transgenic embryos expressing Isl1[ss]:lexA, lexAop:mRuby-caax and tp63:Gal4VP16; UAS:egfp-plcδ-ph (Rasmussen et al., 2015) were mounted as described above and imaged on a ZEISS LSM 980, heated to approximately 28°C, and processed with Airyscan Joint Deconvolution at a maximum of five iterations. Basal skin cells in a rounded state were identified by eye when embryos were 28 – 31 hpf and immediately imaged by time lapse with 5-minute intervals, until daughter basal cells appeared flattened for at least two time points. Axon caliber was measured by line scan analysis, as described above, at 1-μm intervals, excluding regions less than 3 μm from a branch point. Border to border length of axons was measured using the Line tool in ImageJ (Schindelin et al., 2012). Orthogonal projections were generated in ZEN Blue (ZEISS).

### Plotting

All dot plots were created in R. Code can be found at github.com/katieching/CaliberAnalysis. Histograms, dynamics, and pearling plots were created using Microsoft Excel.

### Experimental design and statistical analysis

For descriptive analyses, an appropriate size for the data set was chosen in advance, and images were collected in experimental batches until the predetermined size was exceeded. For statistical comparisons, a small data set was initially collected, and a power analysis was performed to determine the data set size needed to reach a p-value < 0.05. The data set was collected and analyzed before performing final statistical analyses. Data were excluded as necessary for technical reasons, namely image quality that was too poor for line scan analysis, segments where branch points were closer together than the distance indicated for the experiment (e.g. < 5 μm for symmetry analysis), images in which the primary branch caliber was below the limit of resolution for the system (Fig 3, 4), and axons in which > 20% of measurements at the initial time point were below the limit of resolution for the system (Fig 5, 6). Significance of differences in paired data was evaluated by paired permutation test in R. Differences between measurements treated as populations were evaluated by Mann-Whitney test. Analyses performed in R can be found at github.com/katieching/CaliberAnalysis. The sex of animals included in the study was not considered because experiments occurred prior to sex determination.

## Results

### Rohon-Beard (RB) neurons display a variety of calibers.

RB neurons are touch-sensing neurons that differentiate and become functional within two days of development in embryonic zebrafish and other anamniotes (Katz et al., 2021). The peripheral axons of RB neurons can be identified by transmission electron microscopy (TEM) as membrane-bound ovals between the outermost (periderm) skin cells and lower (basal) skin cells in cross section (O’Brien et al., 2012). Using existing TEM data sets (Faas et al., 2012; O’Brien et al., 2012), we identified peripheral axons of RB neurons and trigeminal neurons, closely-related sensory neurons that perform the same touch-sensing function in the head. By measuring the short axis of these axon cross-sections, we found that calibers ranged from approximately 0.084 to 1.459 μm. This wide range prompted us to ask if axon caliber varies within each cell or only between cells, perhaps by sensory subtype (Gau et al., 2013; Palanca et al., 2013; Tuttle et al., 2024).

To determine if axon caliber can vary within an axon arbor, we sought to measure caliber at consistent locations across many cells. To enable absolute measurements of axon caliber, we developed an imaging protocol that paired sparse labeling with super-resolution microscopy. Using transient transgenesis, we generated embryos that expressed a membrane-localized fluorescent protein (EGFP-CAAX) in a single RB neuron in the tail (Fig 1B). We performed time lapse confocal microscopy starting shortly after RB neuron differentiation (20 hours post-fertilization, hpf) and confirmed that branches of the peripheral axon arbor form by growth cone bifurcation as well as collateral sprouting (Sagasti et al., 2005; Haynes et al., 2022), resulting in a highly branched axon arbor within hours of skin entry. To identify a consistent area of measurement, we observed the most proximal branch point in the peripheral arbor at 27 – 30 hpf and found that it typically forms by collateral sprouting (10 out of 10 cells, Fig 1C–C’). In these images, some branches of each arbor appeared to be thick while others appeared to be thin (Fig 1D). This observation suggested that axon caliber can vary within each cell and supports the use of this location and technique for studying axon caliber variation.

### Caliber varies along the length of an axon segment.

**Figure 2:**
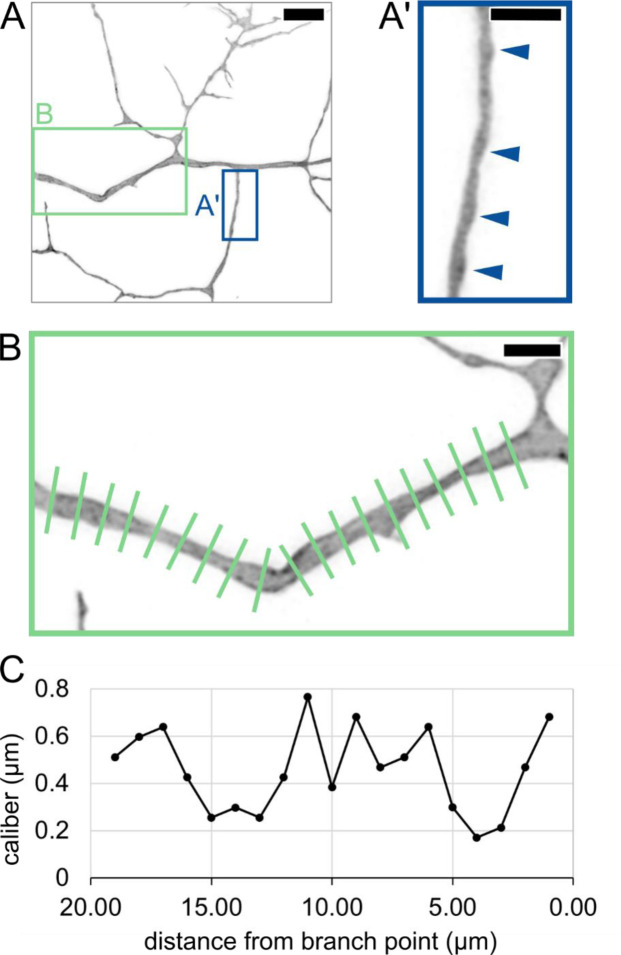
Lengthwise variation in axon caliber **(A)** Image of an RB axon arbor at 29.00 hpf. Blue box shows location of inset in A’. Green box shows location of inset in B. Scale bar: 5 μm. **(A’)** Inset showing axon branch with pearled morphology. Arrowheads: Thicker regions of axon, resembling “pearls.” Scale bar: 2 μm. **(B)** Long axon segment measured by line scan at 1-μm increments. Scale bar: 2 μm. **(C)** Plot of caliber showing variation along the length of axon segment in panel B.

Previous studies have shown that axons are not cylindrical but vary in caliber along their lengths (Greenberg et al., 1990; Bar-Ziv et al., 1999; Pullarkat et al., 2006; Griswold et al., 2024). This phenomenon is sometimes referred to as pearling or varicose morphology. To determine if pearling is observed in live animals, we labeled individual RB neurons and measured axon caliber at 1-μm increments along the length of a segment, shortly after arbor formation began (29 hpf, Fig 2A–B). Variations in axon caliber were visible along the length of all resolvable axon segments, as has been described in mammalian neurons (Greenberg et al., 1990; Griswold et al., 2024). In some instances, axons had distinct pearls (Fig 2A’) and in others, more subtle thick and thin stretches without distinct inflection points (Fig 2C). These observations of lengthwise caliber variation confirm that fish and mammalian neurons share similarities in local axon morphology.

### Sister axon branch calibers are largely independent of each other.

**Figure 3:**
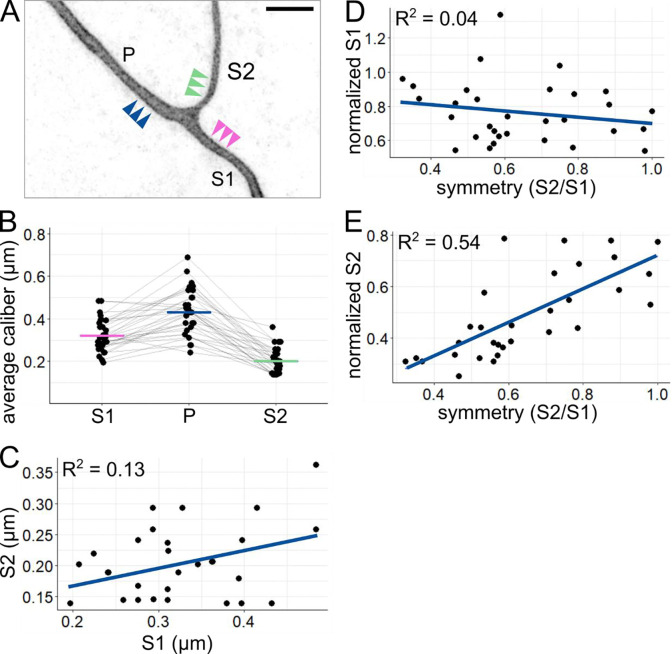
Comparison of axon segments within each arbor **(A)** Example image of proximal RB axon arbor. Arrowheads indicate approximate location of line scans for average caliber measurement. P: primary branch, S1: thicker secondary branch, S2: thinner secondary branch. Scale bar: 5 μm. **(B)** Plot of average axon calibers at the proximal branch point. Grey lines connect data points from the same neuron. Horizontal lines: mean values with colors corresponding to branch categories shown in panel A. n = 31 neurons. **(C)** Plot comparing caliber of sister axon segments shown in panel A. **(D)** Plot of branch symmetry versus normalized S1 caliber (S1/P). **(E)** Plot of branch symmetry versus normalized S2 caliber (S2/P). Blue lines in C, D, and E show linear regression.

Next, we asked how axon caliber varies between different axon segments within a single cell. We labeled individual RB neurons and measured axon caliber near the branch point most proximal to the cell body in embryos 27 – 31 hpf (Fig 3A). The axon segment coming from the spinal cord was designated the primary axon (P), and the axon segments distal to the branch point were designated secondary axons (sister branches designated S1 and S2, the thicker and thinner segments, respectively). Each segment was measured at three locations (3, 4, and 5 μm from the branchpoint), which were averaged to determine the caliber of that axon segment. By measuring these locations, we found that axon segments of different calibers exist within the same RB neuron and that secondary branches were typically thinner than the primary branch from which they arose (Fig 3B, P = 0.43 μm, S1 = 0.32 μm, S2 = 0.20 μm). These observations confirmed that the average caliber of axons is not uniform across the cell.

Because sister segments, S1 and S2, often have different calibers, we sought to assess the relationship between them. We considered two hypotheses: 1) despite variations, each neuron has characteristically thick or thin calibers, and thus S1 and S2 have positively correlated calibers, or 2) S1 and S2 compete for cellular material, and thus their calibers are negatively correlated. To distinguish between these two hypotheses, we compared the S1 and S2 caliber for each neuron. Direct comparison showed a weak relationship between S1 and S2 caliber (Fig 3C, R^2^ = 0.13). This weak correlation suggests that neither hypothesis is strongly supported and that sister branch calibers are largely independent.

In keeping with this interpretation, symmetry across the branch point was weakly correlated with normalized S1 caliber (Fig 3D, R^2^ = 0.04), which we would expect to be strongly correlated if branches compete for cellular material to increase caliber. On the other hand, symmetry is more strongly correlated with normalized S2 caliber (Fig 3E, R^2^ = 0.54), suggesting that symmetry is achieved by the thinner branch becoming thicker, independent of the S1 branch. Together, these results suggest that axon segments can be regulated independently or that their calibers are influenced by local factors.

### RB axon tapering favors afferent axon function.

For an RB neuron to function properly, the cell needs electrical signals to travel efficiently. At the time points measured in Fig 3, the arbor was just a few hours old, functional, and continuing to expand. Already, optimal RB neuron function of transmitting sensory signals from the periphery requires efficient propagation of electrical signals from distal to proximal axon segments (toward the soma). Although tapering is often considered a characteristic unique to dendrites (Craig and Banker, 1994; Jan and Jan, 2010), broader analyses suggest that branched axons may taper as well (Desai-Chowdhry et al., 2022).

**Figure 4:**
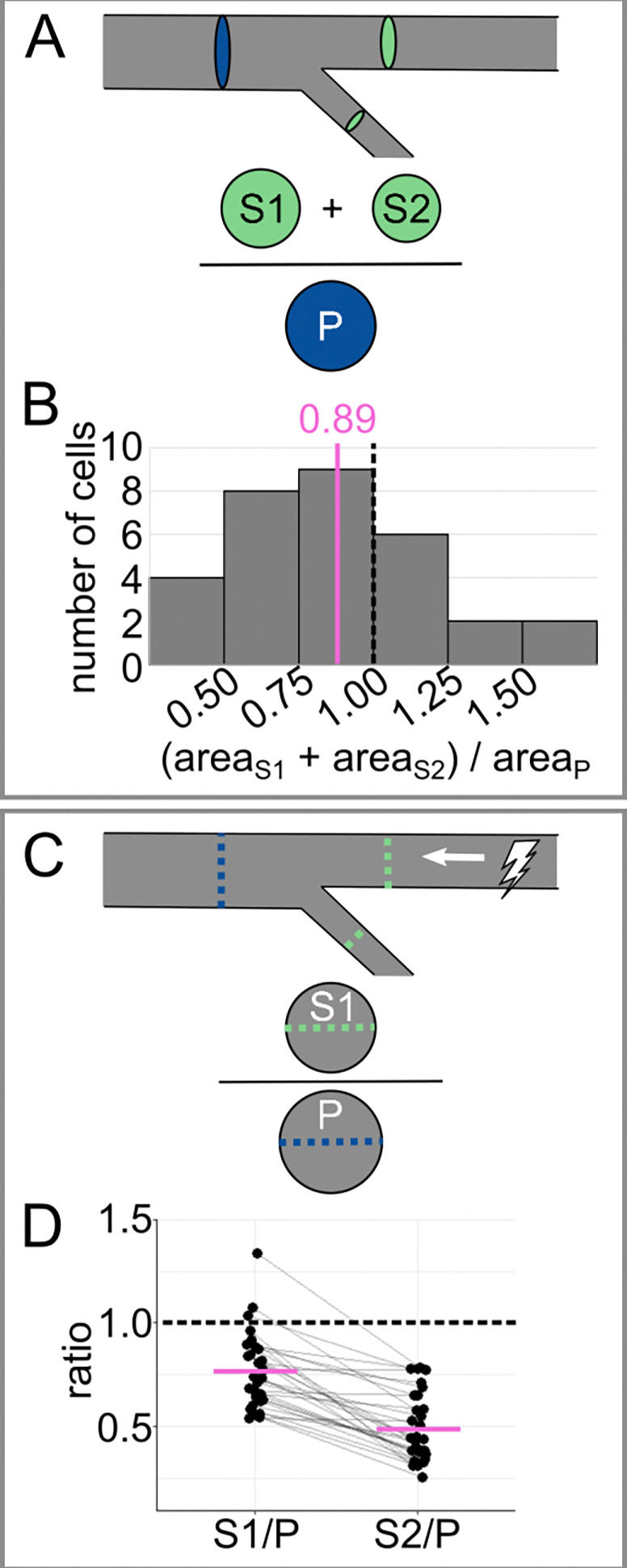
Analysis of tapering across the most proximal branch point **(A)** Diagram of tapering analysis in panel B. Combined cross-sectional area of secondaries (S1 and S2) is divided by the cross-sectional area of the primary (P) to evaluate tapering. **(B)** Histogram of cross-sectional area ratios for data shown in Figure 1. **(C)** Diagram of tapering analysis in panel D. Caliber of each secondary (S1 or S2) is divided by the caliber of the primary (P) to estimate the impact of tapering on action potential conduction velocity. **(D)** Plot of caliber ratios for data shown in Figure 1. Grey lines connect data points from the same neuron. In panels B and D, dotted black lines mark reference value 1.0 (no tapering), and magenta lines mark mean values.

To facilitate comparison of RB axon branching to other systems, we assessed tapering. First, we calculated the cross-sectional area of each primary and secondary branch ($area=\pi^{*}{\left(\frac{1}{2}\text{caliber}\right)}^{2}$). We compared the primary cross-sectional area to the sum of the secondary cross-sectional areas (Fig 4A). On average, the ratio of the combined secondary cross-sectional areas to the primary cross-sectional area was less than 1 (mean of $\frac{{\text{area}}_{S1}+{\text{area}}_{S2}}{{\text{area}}_{P}}=0.888$, Fig 4B). This observation confirms that tapering is not exclusively a characteristic of dendrites and can occur in axons.

The axon arbors of RB neurons contain microtubules that are uniformly plus-end out (Shorey et al., 2021). This observation, coupled with their analogous function to dorsal root ganglion (DRG) neurons, categorizes them as afferent axons, relaying messages from distal regions toward the soma via action potentials. According to cable theory, the speed of action potential propagation increases as axon caliber increases (reviewed by Costa et al., 2018).

To evaluate the interplay between branching and neuron function, we compared the radius scaling ratio between secondary branches and their corresponding primary branches $\frac{{\text{r}}_{S}}{{\text{r}}_{P}}$, Fig 4C). The average ratio for the thicker branch was much larger than for the thinner sister branch $\frac{S1}{P}=0.77\pm 0.033$ SEM, and mean $\frac{S2}{P}=0.49\pm 0.030$ SEM, Fig 4D). In evaluating a branched system, some scaling rules hold for the population average, even when branches are asymmetric (Brummer et al., 2017). Hence, to evaluate the function of the arbor more generally, we combined the measurements for the two branches and found that the average ratio (mean $\frac{S}{P}=0.629\pm 0.0282$ SEM) was similar to what is predicted for a system that optimizes for power dissipation rather than time delay (Desai-Chowdhry et al., 2022). Because RB neuron function relies on messages traveling from distal to proximal (toward the soma), this may suggest that tapering prevents conduction time delay from being a major constraint in neurons with branched afferent axons.

### Axon caliber is highly dynamic.

**Figure 5:**
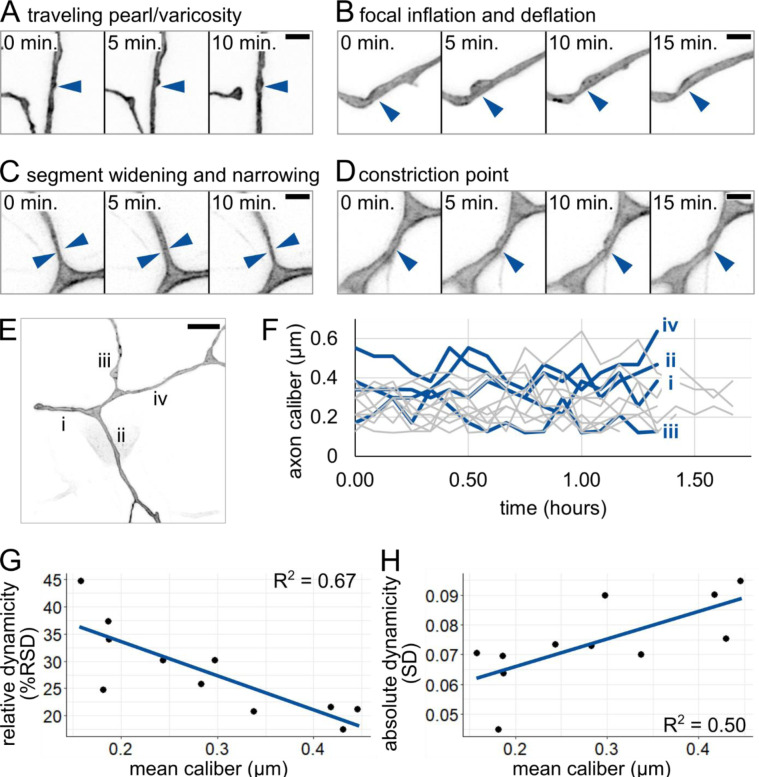
Qualitative and quantitative analysis of caliber dynamics **(A)** Example of possible traveling pearl. Short distance traveled allowed for acquisition despite 5-minute intervals. Arrowhead: initial location of pearl. **(B)** Example of focal inflation and deflation event. Puncta containing EGFP-CAAX accumulate during inflation and are seen exiting during deflation. Arrowhead: location of event. **(C)** Example of segment widening and narrowing. Arrowhead pairs denote width at t = 5 minutes. **(D)** Example of constriction point. Arrowhead: location of event. In panels A-D, scale bars: 2 μm. **(E)** Image of axon arbor in the proximal region. Different segments are labeled from proximal to distal: i-iv. Scale bar: 5 μm. **(F)** Plot of axon caliber dynamics, n = 11 axon segments, N = 5 fish. Blue lines: axon segments shown in panel E. Grey lines: all other axon segments measured. **(G)** Plot of mean caliber versus relative dynamicity (%RSD = (SD / mean caliber) * 100) for all axons shown in panel F. **(H)** Plot of mean caliber versus absolute dynamicity (SD) for all axons shown in panel F. Mean caliber is the average at the given location across all time points. In panels G and H, blue lines: linear regression.

Having documented variability in axon caliber across locations within each neuron, we wondered if calibers change with time. To investigate caliber dynamics, we performed time-lapse imaging in the proximal region of the tail-innervating axon arbors at 28 – 31 hpf. Axon caliber changed in several qualitatively different ways over time. We categorized these dynamic behaviors into four categories: (1) a traveling pearl, instances of bubble-like regions moving along an axon (Fig 5A), (2) focal inflation and deflation, in which a pearl appears and disappears over the course of multiple time points (Fig 5B), (3) segment widening and narrowing, in which an entire segment thickens and thins between time points (Fig 5C), and (4) a constriction point that appears and disappears, sometimes repeatedly (Fig 5D). This variety of dynamic behaviors suggests that several distinct processes likely contribute to axon caliber dynamics.

To assess caliber dynamics quantitatively, we selected several locations within the proximal region of each neuron, each 3 μm away from a branch point, and measured axon caliber at that location over the course of imaging (Fig 5E). We found that axon caliber was highly dynamic (Fig 5F), with standard deviations ranging from 0.045 μm to 0.095 μm, and could vary with a range of up to four-fold at the same location in less than two hours. Hence, axon caliber and local morphology may be more dynamic than has been widely appreciated.

Next, we asked if thin or thick axon segments are more dynamic. To assess relative dynamicity, we calculated relative standard deviation (RSD) for each location by dividing the standard deviation by the average caliber across the movie. Comparing dynamicity across segments revealed that thick axons were less dynamic than thin axons (Fig 5G). There are two possible explanations for this trend: lower relative dynamicity could indicate that fluctuations of similar magnitude occur in thick and thin axons, or that thick axons fluctuate less, in absolute terms. To distinguish between these possibilities, we assessed absolute dynamicity by comparing axon caliber directly to the standard deviation (SD) of measurements observed during the time lapse (Fig 5H). In contrast to the normalized measure, standard deviation and axon caliber correlated positively. Together, these results suggest that thick axons have more or larger absolute fluctuations in caliber, but the fluctuations experienced by thin axons are more dramatic when compared to their average caliber.

### Caliber dynamicity changes over development.

**Figure 6:**
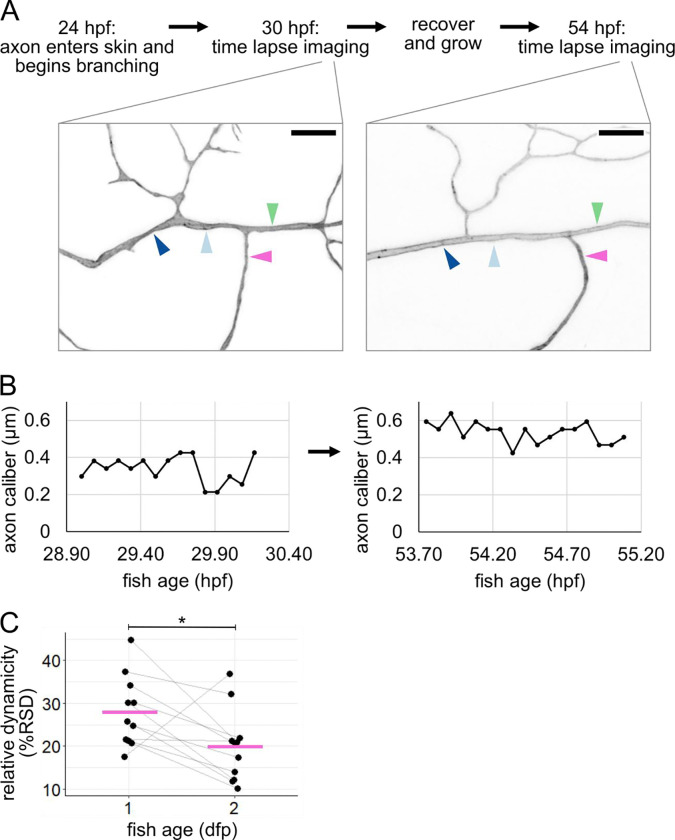
Tracking caliber dynamics over development **(A)** Experimental workflow with example images (same neuron as Figure 2). Arrowheads: locations for line scan measurements. Each location is 3 μm from a branch point and separated from other locations by at least one branch point. Scale bars: 5 μm. **(B)** Representative example plots of caliber dynamics for the same segment at 1 dpf and 2 dpf. **(C)** Plot of relative dynamicity (%RSD) at 1 dpf and 2 dpf (same neurons as Figure 5) n = 11 neurons, N = 5 fish. Grey lines connect data points from the same neuron. Magenta lines: mean values. *p < 0.05.

Because we observed caliber to be highly dynamic early in the life and function of RB neurons, we sought to determine if caliber continues to be dynamic later in development. To do this, we recovered embryos after imaging at 28 – 31 hpf and allowed them to continue developing for an additional 24 hours. These fish were re-mounted at 52 – 55.5 hpf and imaged by time-lapse microscopy again. Although all cells had changed shape and axon arbors had grown, often the locations imaged on the first day could be identified, imaged, and measured again on the second day (Fig 6A–B). We found that all resolvable axons continued to be dynamic. However, dynamicity measured as %RSD decreased from the first to the second day in most axon segments (10 out of 11 axon segments decreased. Mean %RSD = 28% at 1 dpf and 20% at 2 dpf, p = 0.036, Fig 6C). Because some axon segments got thicker (n = 6 out of 11) and some got thinner (n = 5 out of 11), this decline in relative dynamicity is not simply a product of axons becoming universally thicker. Instead, this dynamicity likely reflects developmental changes that occur in the cell and tissue. This could include changes within the cell itself, such as cargo transport (Costa et al., 2020; Wang et al., 2020), or changes to the extracellular environment, such as axon ensheathment (O’Brien et al., 2012; Jiang et al., 2019; Rosa et al., 2023).

### Cellular microenvironment can impact axon caliber.

Based on the qualitative variety of caliber dynamics we observed (Fig 5A–D), we hypothesized that there are multiple contributors to caliber dynamics. Cell-intrinsic effectors that may cause axon caliber to be dynamic, such as cargo transport and contraction of the membrane periodic skeleton, have been described by others (Costa et al., 2020; Wang et al., 2020). However, another possible and often understudied contributor is the cellular microenvironment, including contact with surrounding cells. Because RB axons are embedded in actively developing skin, they are likely subject to deformation caused by morphological changes in surrounding epithelial cells. Specifically, RB peripheral axons grow between two epithelial cell layers, the periderm and basal cells, each of which undergoes rapid expansion at these developmental stages (Fig 1A) (O’Brien et al., 2012).

**Figure 7:**
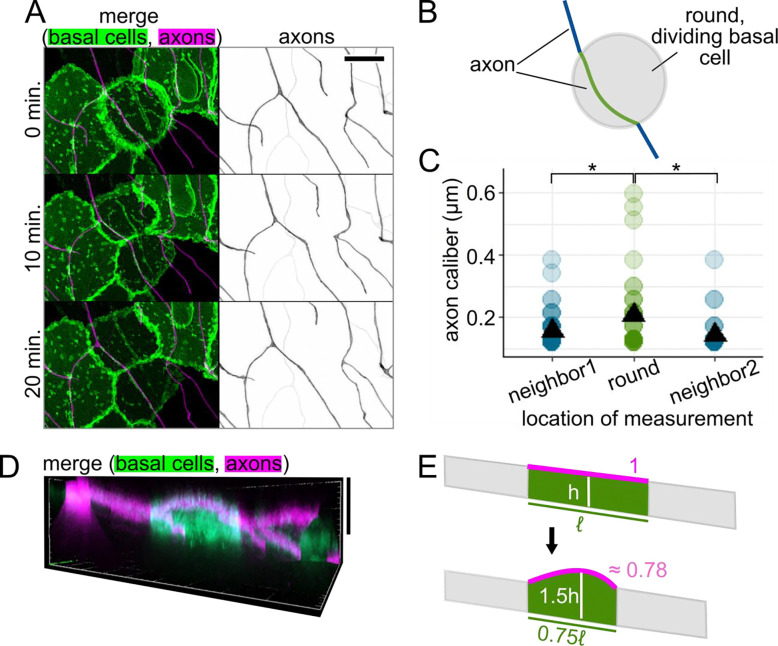
Axon caliber on dividing basal epithelial cells **(A)** Representative images of time lapse showing an RB axon adjacent to a dividing basal cell. Basal cells express EGFP-PLCδ-PH. RB neurons express mRuby-CAAX. Scale bar: 10 μm. **(B)** Diagram of an axon on a dividing basal cell that is rounded while undergoing mitosis. Green line: axon segment in contact with dividing basal cell. Blue lines: axon segments on neighboring, non-dividing basal cells. **(C)** Plot of axon caliber measurements grouped by location, n = 114 measurement locations, N = 4 axons/fish. Green dots: caliber of axon on a dividing basal cell. Blue dots: caliber of axon on neighboring cells to one side (neighbor1) or the other (neighbor2) of the dividing basal cell. Black triangles: mean value. *p-val < 0.05. **(D)** Orthogonal projection of a dividing basal cell, which expresses EGFP-PLCδ-PH and is highlighted against non-dividing basal cells due to a variegated expression pattern. An RB axon is in contact with its apical surface, which is rounded upward. Vertical scale bar: 10 μm. **(E)** Diagram comparing axon path length on a rounded, dividing basal cell to its flattened state as an approximation of how basal cell shape changes may impact tension on the axon. Magenta: approximated, theoretical axon length, estimated as the longest distance across the apical surface in an orthogonal projection. Approximate arc length was calculated based on height and length estimates in orthogonal projections. ℓ: original length of basal cell, h: original height of basal cell.

To determine if changes to the shape or adhesion of adjacent cells can impact the caliber of axons, we performed simultaneous time lapse imaging of axons and the underlying basal cells starting at 28 – 31 hpf. We imaged basal cells using a marker of lipid microdomains under the control of the ΔNp63 promoter (Rasmussen et al., 2015; Rosa et al., 2023). One of the most dramatic and frequent perturbations to the RB axon environment occurs when basal cells round up for mitosis, during which the lipid reporter clearly highlights rounded cells against the field of surrounding, flat basal cells (Fig 7A). We measured caliber at 1-μm intervals along the length of the axon, from a flat neighbor cell, onto the rounded cell, and onto the next flat neighbor cell (Fig 7B). We found that segments of axon on the rounded cell were significantly thicker than segments of the same axon on the non-dividing neighbor cells (caliber = 0.21 μm on rounded cell, 0.16 and 0.14 μm on non-dividing neighbors, p-value = 0.025 and 0.00035, respectively, Fig 7C). This difference contrasts with the two non-dividing neighbor groups, which did not have significantly different calibers, as expected (p-value = 0.059). This observation suggests that changes in the shape or adhesive properties of the dividing cell can impact axon caliber.

### Forces from surrounding cells impact axon morphology.

One reason why axons might be thicker on the rounded cell could be due to changes in tension as the basal cell pulls its borders inward, becoming shorter in planar distance, and rounds up, creating a greater vertical distance (Fig 7D). We used images with the clearest orthogonal projections to obtain estimates of how the dimensions of a dividing basal cell differs from its non-dividing neighbors. Using these estimates, we calculated theoretical changes in axon length as the cell rounds up (Fig 7E). To simplify these calculations, we assumed that the axon is fixed at the cell borders and runs in a straight path across the basal cell surface. We found that the rounding of basal cells resulted in a shorter axon path during division than when cells were flat (path length when round ≈ 0.78 * path length when flat). Assuming that axons are adhered to the non-dividing neighboring cells, these estimates are consistent with the axon being under lower tension on the rounded cell than on a non-dividing neighbor cell.

**Figure 8:**
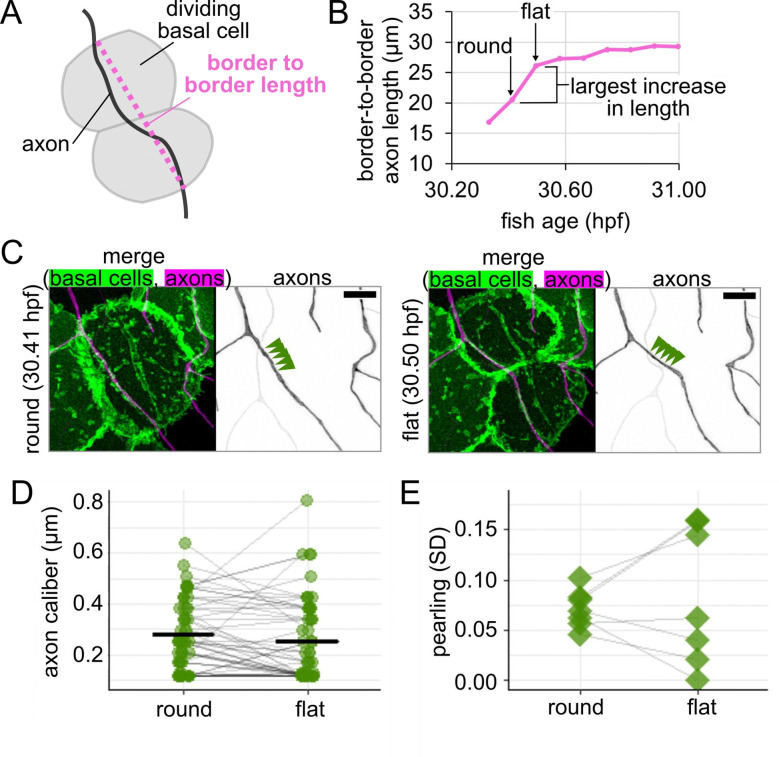
Changes to axon morphology as new basal epithelial cells flatten **(A)** Diagram of how border-to-border length was measured for an axon on a dividing basal cell. **(B)** Example plot of changes in border-to-border axon length during time lapse imaging for axon shown in Fig 7A. Round and flat time points are defined as sequential points (5-min. interval) during which border-to-border length increases the most. **(C)** Images from time points highlighted in panel B. Arrowheads: five measurement locations. Scale bars: 5 μm. **(D)** Plot of axon caliber for all locations that remained on the dividing basal cell or its daughter cells, n = 54 measurement locations, N = 7 axons/fish. Grey lines connect data points from the same location. Black lines: mean values. **(E)** Plot of pearling, defined as SD across all locations, to assess morphology change for axons shown in panel D, N = 7 axons/fish. Grey lines connect data points from the same axon.

Based on our observations of rounded cells and our estimates of length changes, we expected that axon caliber would decline as the daughter basal cells flatten. To test this prediction, we performed time-lapse imaging with 5-minute intervals on the rounded cells, including those in Fig 7C, to see if axon caliber changes as the rounded cell divides and flattens into two daughter cells. For each time point, we measured the distance between the two points where the axon crossed the border of the dividing cell or its daughter cells (Fig 8A). The two time points between which the change in length was greatest were selected, and caliber of the axon on the rounded cell or its daughters was measured at 1-μm increments for those two time points (Fig 8B–C). We paired measurements by location to assess if there was a significant drop in axon caliber between these two time points. Although axon caliber declined from the round to flat time point, on average, this subtle decrease did not reach statistical significance (caliber = 0.28 μm when the basal cell was round and 0.25 μm when the basal cell was flat, p-value = 0.17, n = 54 measurements, N = 7 axons/fish, Fig 8D). This contrasts with the portions of the axon that were on non-dividing neighbor cells, where the difference could have easily occurred by random chance (p-value = 0.94, n = 37 measurements). This result suggests that the decrease in average axon caliber as the underlying basal cell flattened is small and likely not meaningful.

Despite the lack of change in average caliber, visual assessment of the videos appeared to show a change in morphology during basal cell flattening (Fig 7A, 8C). Tension may be an important parameter in pearling (Bar-Ziv et al., 1999; Pullarkat et al., 2006; Griswold et al., 2024), so we wondered if thick regions (i.e. pearls) and thin regions (i.e. connectors) undergo different changes during basal cell division.

To assess morphological changes to axons during basal cell division, we calculated pearling as the standard deviation of measurements taken along the length of each axon for the rounded time point and for the flat time point. By pairing values from each movie, we found that one subset of axons became more pearled (increased SD) and the rest became less pearled (decreased SD), resulting in two groups of cellular responses to flattening (Fig 8E). This heterogeneity suggests that either 1) different responses arise from heterogeneity among the RB neuron population, or 2) our 5-minute intervals do not provide sufficient time resolution to capture cellular responses to tension, which may occur in less than 10 minutes (Sinha et al., 2011; Sitarska and Diz-Muñoz, 2020). Regardless of the reason, these results demonstrate that axon morphology changes with basal cell flattening, though additional complexities remain to be explored. As different portions of an axon contact different basal cells, these findings suggest that morphological changes in basal cells may account for some of the observed spatial and temporal variations in RB axon caliber.

## Discussion

### Tapered architecture in branched sensory axons

We have found that the calibers of axon segments in the same neuron can be largely independent of one another, highly dynamic, and likely shaped by multiple determinants, including the extracellular environment. Variation in axon caliber had previously been observed along short stretches of fixed axons in rodent neurons (Greenberg et al., 1990; Griswold et al., 2024), but our results expand upon these findings by revealing variation between larger axon segments, across branch points, and over time.

The cutaneous axon endings of touch-sensing neurons, including trigeminal, dorsal root ganglion (DRG), and RB neurons, share characteristics with both dendrites and axons. Like dendrites, they detect stimuli from the periphery and relay signals towards the cell body, but, like axons, their microtubules are oriented plus-end-out (Shorey et al., 2021) and presumably generate action potentials. We found that the branched axon arbors of RB neurons taper across the most proximal branch point, becoming narrower in caliber and cross-sectional area further from the soma (Fig 4). This feature is often regarded as a characteristic of dendrites, not of axons (Craig and Banker, 1994; Jan and Jan, 2010). Notably, tapering favors afferent action potential conduction, suggesting that tapering may be most aligned with direction of functional signal propagation and is likely not determined by microtubule polarity. Other studies have also shown that tapering can occur in both dendrites and axons of other types of neurons (Desai-Chowdhry et al., 2022, 2023), demonstrating that the degree to which a neuronal process tapers could be more closely tied to optimization pressures than its identity as an axon or dendrite.

In comparing sister axon branches, we hypothesized that sister branches are related to each other, resulting from either cell-wide effects or a competition for cellular materials. Instead, we found that their calibers are largely independent of each other, despite being in close proximity and sharing a cytoplasm. Interestingly, we observed varying degrees of symmetry, ranging from pairs of sister branches with the same caliber to highly asymmetrical pairs, where one secondary branch is much thicker than the other. One reasonable hypothesis to explain this observation could be that branches of different symmetry form by different mechanisms. For example, highly asymmetric branches might have formed by collateral sprouting and symmetric branches by growth bifurcation. However, we found a full spectrum of symmetry values, not a bimodal distribution, arguing against this hypothesis (Fig 3D–E). Moreover, though bifurcations are common in RB arbor outgrowth (Haynes et al., 2022) the first branch point often, if not always, forms as a collateral (Fig 1C). Thus, collateral sprouting can give rise to segments of a variety of calibers within a few hours of formation, highlighting the malleability of axon caliber.

### Mechanisms of caliber variation over time and space

Time lapse imaging of axon caliber revealed multiple qualitatively different types of dynamics (Fig 5A–D), which we refer to as traveling pearls, focal inflation and deflation, segment widening and narrowing, and constriction points. This variety suggests that there are likely multiple contributors to variations in axon caliber, including both cell-intrinsic and cell-extrinsic mechanisms.

Cell-intrinsic determinants of axon caliber, such as the cytoskeleton, have been studied to varying degrees. One of the best-known caliber determinants is neurofilaments, a type of intermediate filament specific to the nervous system. Increased neurofilament abundance increases axon caliber in mammalian and bird neurons (Ohara et al., 1993; Sakaguchi et al., 1993; Eyer and Peterson, 1994; Zhu et al., 1997; Elder et al., 1998; Kriz et al., 2000; Sainio et al., 2021). Neurofilaments are also highly dynamic. Differences in transport, differential modifications, and dynamics (e.g. folding, severing, annealing) of neurofilaments are all possible ways in which these filaments might contribute to both local regulation and axon caliber dynamics within a cell (Çolakoğlu and Brown, 2009; Boyer et al., 2022; Uchida et al., 2023).

Another cytoskeletal candidate for cell-intrinsic effectors of caliber variation is the more recently discovered membrane periodic skeleton (MPS), regularly-spaced actin rings connected by spectrin tetramers that run the length of axons and dendrites (Xu et al., 2013; He et al., 2016). These rings expand when large cargo such as mitochondria and lysosomes pass through, and altering myosin contractility changes caliber and retrograde trafficking (Leite et al., 2016; Costa et al., 2020; Wang et al., 2020). Work by Griswold et al. demonstrated that myosin contractility can impact pearling (Griswold et al., 2024), but whether the MPS influences variation between branches and caliber dynamics remains to be explored. Observations of cargo transport through the MPS (Costa et al., 2020; Wang et al., 2020), as well as fixed observations that large cargo is often found within pearls (Greenberg et al., 1990), suggest that intracellular cargo transport along microtubules may also contribute to axon caliber dynamics.

Membrane composition is another non-cytoskeletal, cell-intrinsic candidate for causing caliber variation. Griswold et al. found that membrane fluidity can change which size and shape of pearls are most energetically favorable, thereby changing axon morphology (Griswold et al., 2024). Given the dynamic nature of caliber in vivo, we hypothesize that changes to membrane composition could also change caliber dynamics.

Damage can also influence axon morphology. Markedly pearled or varicose morphologies, sometimes referred to as beading or swelling, often form in damaged axons (Kerschensteiner et al., 2005; Williams et al., 2014; Datar et al., 2019; Shao et al., 2020). Indeed, damaged RB axons, themselves, become dramatically more beaded just before degeneration (Martin et al., 2010). Our observations of variation in caliber in this study were made in anesthetized, healthy embryos and are unlikely to result from axon damage, as neurons remained intact, even after time lapse microscopy. Similarity between these phenomena in healthy and damaged axons may reflect related processes if the healthy malleability of axon caliber is pushed to extremes upon insult.

Although most attention has focused on potential intrinsic determinants for axon caliber variation, we considered the possibility that extrinsic influences from surrounding cells might affect axon caliber. RB axon arbors are embedded in a developing epidermis, which undergoes growth and morphogenetic changes. We found that changes in tension caused by the movement of cells adjacent to axons are candidates for cell-extrinsic regulators of axon caliber. RB axons span tens or even hundreds of micrometers in length and branch to cover large areas of the skin. Hence, each segment of an axon contacts different basal cells, which may each impact the local axon caliber independently.

Basal cells can undergo many morphological changes to alter the axon’s environment. One important change is ensheathment ((O’Brien et al., 2012), Fig 1A), which increases between 1 and 2 dpf (Jiang et al., 2019; Rosa et al., 2023), possibly contributing to differences in dynamicity between these two developmental time points (Fig 6C). The idea that ensheathment may alter axon caliber is further supported by the observation that myelinated portions of an axon are thicker than the nodes of Ranvier (Rydmark, 1981; Swärd et al., 1995).

Another morphological change in basal cells is rounding for division, which we found changes axon caliber and morphology (Fig 7,8). Division events likely vary in frequency throughout embryonic development, which may further contribute to differences in caliber dynamicity (Fig 6C). Additionally, axons may experience end-to-end stretch as embryos lengthen. Finally, although our studies were in embryonic zebrafish, when the environment surrounding these neurons is a rapidly expanding bilayered epidermis, dynamics are unlikely to cease in adulthood, since the stratified epidermis in both fish and mammals is constantly renewing as basal cells differentiate into higher strata. How caliber variations and dynamics differ in this stratified system remains to be explored. Together, our results suggest that lengthwise variation, local variations in average caliber, and caliber dynamics may be influenced, in part, by changes in the microenvironment that surrounds axons.

## Significance Statement

Axon caliber directly influences how quickly neurons send messages to other cells and likely plays a role in the overall health of neurons. In the peripheral nervous system, where neurons cover particularly long distances, cell shape can determine whether an animal successfully executes behaviors such as an escape response. We found that axon caliber can vary between locations within the same cell, and that it is highly dynamic. Taking these variations into account may allow neuroscientists to better estimate transmission speeds for cells in neural circuits. Additionally, we found that axon caliber is distorted when nearby cells change their shape. Thus, the cellular microenvironment is one of potentially many contributors to caliber dynamics, broadening our view of axon caliber determinants.

## References

[R1] Bar-Ziv R, Tlusty T, Moses E, Safran SA, Bershadsky A (1999) Pearling in cells: a clue to understanding cell shape. Proc Natl Acad Sci U S A 96:10140–10145. 10468576 10.1073/pnas.96.18.10140 PMC17856

[R2] Boyer NP, Julien J-P, Jung P, Brown A (2022) Neurofilament Transport Is Bidirectional In Vivo. eNeuro 9 Available at: 10.1523/ENEURO.0138-22.2022. PMC9410771 35896389

[R3] Brummer AB, Savage VM, Enquist BJ (2017) A general model for metabolic scaling in self-similar asymmetric networks. PLoS Comput Biol 13:e1005394. 28319153 10.1371/journal.pcbi.1005394 PMC5378416

[R4] Çolakoğlu G, Brown A (2009) Intermediate filaments exchange subunits along their length and elongate by end-to-end annealing. The Journal of Cell Biology 185:769–777 Available at: 10.1083/jcb.200809166. 19468066 PMC2711597

[R5] Costa AR, Pinto-Costa R, Sousa SC, Sousa MM (2018) The Regulation of Axon Diameter: From Axonal Circumferential Contractility to Activity-Dependent Axon Swelling. Front Mol Neurosci 11:319. 30233318 10.3389/fnmol.2018.00319 PMC6131297

[R6] Costa AR, Sousa SC, Pinto-Costa R, Mateus JC, Lopes CD, Costa AC, Rosa D, Machado D, Pajuelo L, Wang X, Zhou F-Q, Pereira AJ, Sampaio P, Rubinstein BY, Mendes Pinto I, Lampe M, Aguiar P, Sousa MM (2020) The membrane periodic skeleton is an actomyosin network that regulates axonal diameter and conduction. Elife 9 Available at: 10.7554/eLife.55471. PMC7105375 32195665

[R7] Craig AM, Banker G (1994) Neuronal polarity. Annu Rev Neurosci 17:267–310. 8210176 10.1146/annurev.ne.17.030194.001411

[R8] Datar A, Ameeramja J, Bhat A, Srivastava R, Mishra A, Bernal R, Prost J, Callan-Jones A, Pullarkat PA (2019) The roles of microtubules and membrane tension in axonal beading, retraction, and atrophy. Biophys J 117:880–891. 31427070 10.1016/j.bpj.2019.07.046 PMC6731471

[R9] Desai-Chowdhry P, Brummer AB, Mallavarapu S, Savage VM (2023) Neuronal branching is increasingly asymmetric near synapses, potentially enabling plasticity while minimizing energy dissipation and conduction time. J R Soc Interface 20:20230265. 37669695 10.1098/rsif.2023.0265 PMC10480011

[R10] Desai-Chowdhry P, Brummer AB, Savage VM (2022) How axon and dendrite branching are guided by time, energy, and spatial constraints. Sci Rep 12:20810. 36460669 10.1038/s41598-022-24813-2 PMC9718790

[R11] Dollé J-P, Jaye A, Anderson SA, Ahmadzadeh H, Shenoy VB, Smith DH (2018) Newfound sex differences in axonal structure underlie differential outcomes from in vitro traumatic axonal injury. Exp Neurol 300:121–134. 29104114 10.1016/j.expneurol.2017.11.001 PMC6495524

[R12] Elder GA, Friedrich VL Jr, Kang C, Bosco P, Gourov A, Tu PH, Zhang B, Lee VM, Lazzarini RA (1998) Requirement of heavy neurofilament subunit in the development of axons with large calibers. J Cell Biol 143:195–205. 9763431 10.1083/jcb.143.1.195 PMC2132822

[R13] Eyer J, Peterson A (1994) Neurofilament-deficient axons and perikaryal aggregates in viable transgenic mice expressing a neurofilament-beta-galactosidase fusion protein. Neuron 12:389–405. 8110465 10.1016/0896-6273(94)90280-1

[R14] Faas FGA, Avramut MC, van den Berg BM, Mommaas AM, Koster AJ, Ravelli RBG (2012) Virtual nanoscopy: generation of ultra-large high resolution electron microscopy maps. J Cell Biol 198:457–469. 22869601 10.1083/jcb.201201140 PMC3413355

[R15] Gau P, Poon J, Ufret-Vincenty C, Snelson CD, Gordon SE, Raible DW, Dhaka A (2013) The zebrafish ortholog of TRPV1 is required for heat-induced locomotion. J Neurosci 33:5249–5260. 23516290 10.1523/JNEUROSCI.5403-12.2013 PMC3893356

[R16] Greenberg MM, Leitao C, Trogadis J, Stevens JK (1990) Irregular geometries in normal unmyelinated axons: a 3D serial EM analysis. J Neurocytol 19:978–988. 2292722 10.1007/BF01186825

[R17] Griswold JM, Bonilla-Quintana M, Pepper R, Lee CT, Raychaudhuri S, Ma S, Gan Q, Syed S, Zhu C, Bell M, Suga M, Yamaguchi Y, Chéreau R, Valentin Nägerl U, Knott G, Rangamani P, Watanabe S (2024) Membrane mechanics dictate axonal pearls-on-a-string morphology and function. Nat Neurosci Available at: https://pubmed.ncbi.nlm.nih.gov/3962/. 39623218 10.1038/s41593-024-01813-1 PMC11706780

[R18] Haynes EM, Burnett KH, He J, Jean-Pierre MW, Jarzyna M, Eliceiri KW, Huisken J, Halloran MC (2022) KLC4 shapes axon arbors during development and mediates adult behavior. Elife 11 Available at: https://pubmed.ncbi.nlm.nih.gov/36222498/ [Accessed November 22, 2024]. 10.7554/eLife.74270 PMC9596160 36222498

[R19] He J, Zhou R, Wu Z, Carrasco MA, Kurshan PT, Farley JE, Simon DJ, Wang G, Han B, Hao J, Heller E, Freeman MR, Shen K, Maniatis T, Tessier-Lavigne M, Zhuang X (2016) Prevalent presence of periodic actin-spectrin-based membrane skeleton in a broad range of neuronal cell types and animal species. Proc Natl Acad Sci U S A 113:6029–6034. 27162329 10.1073/pnas.1605707113 PMC4889411

[R20] Hursh JB (1939) Conduction velocity and diameter of nerve fibers. Am J Physiol 127:131–139.

[R21] Jan Y-N, Jan LY (2010) Branching out: mechanisms of dendritic arborization. Nat Rev Neurosci 11:316–328. 20404840 10.1038/nrn2836 PMC3079328

[R22] Jiang N, Rasmussen JP, Clanton JA, Rosenberg MF, Luedke KP, Cronan MR, Parker ED, Kim H-J, Vaughan JC, Sagasti A, Parrish JZ (2019) A conserved morphogenetic mechanism for epidermal ensheathment of nociceptive sensory neurites. Elife 8 Available at: 10.7554/eLife.42455. PMC6450671 30855229

[R23] Katz HR, Menelaou E, Hale ME (2021) Morphological and physiological properties of Rohon-Beard neurons along the zebrafish spinal cord. J Comp Neurol 529:1499–1515. 32935362 10.1002/cne.25033

[R24] Kerschensteiner M, Schwab ME, Lichtman JW, Misgeld T (2005) In vivo imaging of axonal degeneration and regeneration in the injured spinal cord. Nat Med 11:572–577. 15821747 10.1038/nm1229

[R25] Kriz J, Zhu Q, Julien JP, Padjen AL (2000) Electrophysiological properties of axons in mice lacking neurofilament subunit genes: disparity between conduction velocity and axon diameter in absence of NF-H. Brain Res 885:32–44. 11121527 10.1016/s0006-8993(00)02899-7

[R26] Leite SC, Sampaio P, Sousa VF, Nogueira-Rodrigues J, Pinto-Costa R, Peters LL, Brites P, Sousa MM (2016) The Actin-Binding Protein α-Adducin Is Required for Maintaining Axon Diameter. Cell Rep 15:490–498. 27068466 10.1016/j.celrep.2016.03.047 PMC4838511

[R27] Martin SM, O’Brien GS, Portera-Cailliau C, Sagasti A (2010) Wallerian degeneration of zebrafish trigeminal axons in the skin is required for regeneration and developmental pruning. Development 137:3985–3994. 21041367 10.1242/dev.053611 PMC2976282

[R28] O’Brien GS, Rieger S, Wang F, Smolen GA, Gonzalez RE, Buchanan J, Sagasti A (2012) Coordinate development of skin cells and cutaneous sensory axons in zebrafish. J Comp Neurol 520:816–831. 22020759 10.1002/cne.22791 PMC4299821

[R29] Ochoa-Espinosa A, Affolter M (2012) Branching morphogenesis: from cells to organs and back. Cold Spring Harb Perspect Biol 4:a008243–a008243. 22798543 10.1101/cshperspect.a008243 PMC3475165

[R30] Ohara O, Gahara Y, Miyake T, Teraoka H, Kitamura T (1993) Neurofilament deficiency in quail caused by nonsense mutation in neurofilament-L gene. J Cell Biol 121:387–395. 8468353 10.1083/jcb.121.2.387 PMC2200107

[R31] Palanca AMS, Lee S-L, Yee LE, Joe-Wong C, Trinh LA, Hiroyasu E, Husain M, Fraser SE, Pellegrini M, Sagasti A (2013) New transgenic reporters identify somatosensory neuron subtypes in larval zebrafish. Dev Neurobiol 73:152–167. 22865660 10.1002/dneu.22049 PMC3541445

[R32] Perge JA, Niven JE, Mugnaini E, Balasubramanian V, Sterling P (2012) Why do axons differ in caliber? J Neurosci 32:626–638. 22238098 10.1523/JNEUROSCI.4254-11.2012 PMC3571697

[R33] Pullarkat PA, Dommersnes P, Fernández P, Joanny J-F, Ott A (2006) Osmotically driven shape transformations in axons. Phys Rev Lett 96:048104. 16486900 10.1103/PhysRevLett.96.048104

[R34] Rasmussen JP, Sack GS, Martin SM, Sagasti A (2015) Vertebrate epidermal cells are broad-specificity phagocytes that clear sensory axon debris. J Neurosci 35:559–570. 25589751 10.1523/JNEUROSCI.3613-14.2015 PMC4293411

[R35] Rosa JB, Nassman KY, Sagasti A (2023) Sensory axons induce epithelial lipid microdomain remodeling and determine the distribution of junctions in the epidermis. Mol Biol Cell 34:ar5. 36322392 10.1091/mbc.E22-09-0396 PMC9816649

[R36] Rydmark M (1981) Nodal axon diameter correlates linearly with internodal axon diameter in spinal roots of the cat. Neurosci Lett 24:247–250. 7279292 10.1016/0304-3940(81)90165-8

[R37] Sagasti A, Guido MR, Raible DW, Schier AF (2005) Repulsive interactions shape the morphologies and functional arrangement of zebrafish peripheral sensory arbors. Curr Biol 15:804–814. 15886097 10.1016/j.cub.2005.03.048

[R38] Sainio MT, Rasila T, Molchanova SM, Järvilehto J, Torregrosa-Muñumer R, Harjuhaahto S, Pennonen J, Huber N, Herukka S-K, Haapasalo A, Zetterberg H, Taira T, Palmio J, Ylikallio E, Tyynismaa H (2021) Neurofilament Light Regulates Axon Caliber, Synaptic Activity, and Organelle Trafficking in Cultured Human Motor Neurons. Front Cell Dev Biol 9:820105. 35237613 10.3389/fcell.2021.820105 PMC8883324

[R39] Sakaguchi T, Okada M, Kitamura T, Kawasaki K (1993) Reduced diameter and conduction velocity of myelinated fibers in the sciatic nerve of a neurofilament-deficient mutant quail. Neurosci Lett 153:65–68. 8510825 10.1016/0304-3940(93)90078-y

[R40] Schindelin J, Arganda-Carreras I, Frise E, Kaynig V, Longair M, Pietzsch T, Preibisch S, Rueden C, Saalfeld S, Schmid B, Tinevez J-Y, White DJ, Hartenstein V, Eliceiri K, Tomancak P, Cardona A (2012) Fiji: an open-source platform for biological-image analysis. Nat Methods 9:676–682. 22743772 10.1038/nmeth.2019 PMC3855844

[R41] Shao X, Sørensen MH, Xia X, Fang C, Hui TH, Chang RCC, Chu Z, Lin Y (2020) Beading of injured axons driven by tension- and adhesion-regulated membrane shape instability. J R Soc Interface 17:20200331.

[R42] Shorey M, Rao K, Stone MC, Mattie FJ, Sagasti A, Rolls MM (2021) Microtubule organization of vertebrate sensory neurons in vivo. Dev Biol Available at: 10.1016/j.ydbio.2021.06.007. PMC8364508 34147472

[R43] Sinha B, Köster D, Ruez R, Gonnord P, Bastiani M, Abankwa D, Stan RV, Butler-Browne G, Vedie B, Johannes L, Morone N, Parton RG, Raposo G, Sens P, Lamaze C, Nassoy P (2011) Cells respond to mechanical stress by rapid disassembly of caveolae. Cell 144:402–413. 21295700 10.1016/j.cell.2010.12.031 PMC3042189

[R44] Sitarska E, Diz-Muñoz A (2020) Pay attention to membrane tension: Mechanobiology of the cell surface. Curr Opin Cell Biol 66:11–18. 32416466 10.1016/j.ceb.2020.04.001 PMC7594640

[R45] Swärd C, Berthold CH, Nilsson-Remahl I, Rydmark M (1995) Axonal constriction at Ranvier’s node increases during development. Neurosci Lett 190:159–162. 7637883 10.1016/0304-3940(95)11528-5

[R46] Tuttle AM, Miller LN, Royer LJ, Wen H, Kelly JJ, Calistri NL, Heiser LM, Nechiporuk AV (2024) Single-Cell Analysis of Rohon-Beard Neurons Implicates Fgf Signaling in Axon Maintenance and Cell Survival. J Neurosci 44 Available at: 10.1523/JNEUROSCI.1600-23.2024. PMC11026351 38423763

[R47] Uchida A, Peng J, Brown A (2023) Regulation of neurofilament length and transport by a dynamic cycle of phospho-dependent polymer severing and annealing. Mol Biol Cell 34:ar68. 36989035 10.1091/mbc.E23-01-0024 PMC10295484

[R48] Wang F, Julien DP, Sagasti A (2013) Journey to the skin: Somatosensory peripheral axon guidance and morphogenesis. Cell Adh Migr 7:388–394. 23670092 10.4161/cam.25000 PMC3739816

[R49] Wang T, Li W, Martin S, Papadopulos A, Joensuu M, Liu C, Jiang A, Shamsollahi G, Amor R, Lanoue V, Padmanabhan P, Meunier FA (2020) Radial contractility of actomyosin rings facilitates axonal trafficking and structural stability. J Cell Biol 219 Available at: 10.1083/jcb.201902001. PMC7199852 32182623

[R50] Williams PR, Marincu B-N, Sorbara CD, Mahler CF, Schumacher A-M, Griesbeck O, Kerschensteiner M, Misgeld T (2014) A recoverable state of axon injury persists for hours after spinal cord contusion in vivo. Nat Commun 5:5683. 25511170 10.1038/ncomms6683

[R51] Xu K, Zhong G, Zhuang X (2013) Actin, spectrin, and associated proteins form a periodic cytoskeletal structure in axons. Science 339:452–456. 23239625 10.1126/science.1232251 PMC3815867

[R52] Zhu Q, Couillard-Després S, Julien JP (1997) Delayed maturation of regenerating myelinated axons in mice lacking neurofilaments. Exp Neurol 148:299–316. 9398473 10.1006/exnr.1997.6654

